# SF-12 or SF-36 in pituitary disease? Toward concise and comprehensive patient-reported outcomes measurements

**DOI:** 10.1007/s12020-020-02384-4

**Published:** 2020-06-19

**Authors:** Merel van der Meulen, Amir H. Zamanipoor Najafabadi, Daniel J. Lobatto, Cornelie D. Andela, Thea P. M. Vliet Vlieland, Alberto M. Pereira, Wouter R. van Furth, Nienke R. Biermasz

**Affiliations:** 1 Department of Medicine, Division of Endocrinology, Pituitary Center and Center for Endocrine Tumors, Leiden University Medical Center, Leiden, The Netherlands; 2 University Neurosurgical Center Holland, Leiden University Medical Center, Haaglanden Medical Center and Haga Teaching Hospital, Leiden/The Hague, The Netherlands; 3 Department of Orthopedics, Rehabilitation and Physical Therapy, Leiden University Medical Center, Leiden, The Netherlands

**Keywords:** Pituitary tumor, Health-related quality of life, Short Form-36, Short Form-12, Patient-reported outcome measure

## Abstract

**Purpose:**

Pituitary diseases severely affect patients’ health-related quality of life (HRQoL). The most frequently used generic HRQoL questionnaire is the Short Form-36 (SF-36). The shorter 12-item version (SF-12) can improve efficiency of patient monitoring. This study aimed to determine whether SF-12 can replace SF-36 in pituitary care.

**Methods:**

In a longitudinal cohort study (August 2016 to December 2018) among 103 endoscopically operated adult pituitary tumor patients, physical and mental component scores (PCS and MCS) of SF-36 and SF-12 were measured preoperatively, and 6 weeks and 6 months postoperatively. Chronic care was assessed with a cross-sectional study (*N* = 431). Mean differences and agreement between SF-36 and SF-12 change in scores (preoperative vs. 6 months) were assessed with intraclass correlation coefficients (ICC) and limits of agreement, depicting 95% of individual patients.

**Results:**

In the longitudinal study, mean differences between change in SF-36 and SF-12 scores were 1.4 (PCS) and 0.4 (MCS) with fair agreement for PCS (ICC = 0.546) and substantial agreement for MCS (ICC = 0.931). For 95% of individual patients, the difference between change in SF-36 and SF-12 scores varied between −14.0 and 16.9 for PCS and between −7.8 and 8.7 for MCS. Cross-sectional results showed fair agreement for PCS (ICC = 0.597) and substantial agreement for MCS (ICC = 0.943).

**Conclusions:**

On a group level, SF-12 can reliably reproduce MCS in pituitary patients, although PCS is less well correlated. However, individual differences between SF-36 and SF-12 can be large. For pituitary diseases, alternative strategies are needed for concise, but comprehensive patient-reported outcome measurement.

## Introduction

Pituitary/sellar tumors are rare, with a prevalence of 78–94 per 100,000 individuals [1]. Both the tumor and its treatment may cause short- and long-term sequelae [2, 3]. Patients may suffer from symptoms due to compression of local critical structures such as the optic nerve [3], and characteristic symptoms in case of hormone excess or deficiency, such as infertility and hypogonadism in prolactinoma [4, 5], and musculoskeletal, cardiovascular, and metabolic abnormalities in acromegaly and Cushing’s disease [6, 7]. Moreover, both functioning and nonfunctioning tumors frequently cause cognitive and psychological symptoms such as mental fatigue, emotional instability, loss of libido, and depressive symptoms [8, 9]. As a result of this complex multisystem morbidity, pituitary/sellar diseases profoundly affect patients’ general health-related quality of life (HRQoL), which generally remains impaired even long after biomedical control [8–10].

Since discrepancies may exist between patients’ perspective on their HRQoL and the more objective clinician-reported outcome measures [11], patient-reported outcome measures (PROMs) are increasingly used both in clinical monitoring, and as outcome measures in clinical trials [8]. Besides disease-specific PROMs, PROMs assessing general HRQoL are used frequently [8], providing the opportunity to compare different disease populations. The Short Form-36 (SF-36) [12] is the most frequently used generic PROM in patients with a pituitary/sellar tumor [8]. This questionnaire consists of 36 questions covering eight domains of health and wellbeing with corresponding subscales, which are used to estimate a physical (PCS) and a mental component score (MCS). A shorter version, the Short Form-12 (SF-12) [13], has been developed, comprising 12 items of the SF-36 that can be used to calculate the PCS and MCS, omitting the subscale scores. The SF-12 has been studied in different patient populations and has shown strong correlations with the SF-36 [14–20] but has not been evaluated in pituitary diseases.

Due to the wide range of local and systemic symptoms, but also characteristic ‘endocrine’ symptoms caused by pituitary/sellar tumors, multiple disease-specific or symptom-specific PROMs should be used to comprehensively measure outcomes relevant for pituitary patients, together with a generic PROM allowing for comparison with other diseases [21]. To increase efficiency and to reduce the patient burden of completing these questionnaires, it would be valuable to investigate whether the number of questions can be reduced, whilst maintaining the capacity to reliably monitor HRQoL in patients with pituitary/sellar disease. Therefore, the aim of this study was to determine whether the SF-12 can be used instead of the SF-36 to assess the PCS and MCS in the monitoring of pituitary/sellar diseases.

## Methods

### Study design

For the analyses, data of two previously published cohorts [21, 22] were used. The first study was a longitudinal cohort of consecutive patients treated surgically for a pituitary/sellar tumor between August 2016 and December 2018 [21], who completed multiple PROMs before, and 6 weeks and 6 months after surgery. The second cohort was a large cross-sectional study performed in a chronic care setting [22], which was used to further validate our results. This cohort consisted of pituitary patients after a median of 13.0 years since diagnosis, recruited between September 2016 and March 2017. Both studies were performed at the Leiden University Medical Center, a Dutch tertiary referral center for patients with pituitary/sellar disease, and were approved by the institutional ethical committee (p16.091, p12.067).

### Patient population

For the longitudinal cohort study, all consecutive patients, ≥18 years, and scheduled for endoscopic transsphenoidal resection of a pituitary/sellar tumor were eligible. For the cross-sectional study, we approached all patients with a history of a pituitary/sellar tumor, aged ≥ 18 years, and under active follow-up at our center. Exclusion criteria included a follow-up of <6 months, insufficient Dutch language skills, an incapacity to complete the questionnaires, and living abroad. For both studies, eligible patients were invited to participate by letter, and were enrolled after informed consent.

### Data collection

#### Baseline characteristics

For the longitudinal study, the baseline characteristics collected from patient charts included age, sex, marital status, education level, tumor type, size, and invasion, date of diagnosis, prior treatment of the tumor, preoperative pituitary function, visual functioning, and cerebral nerve deficits, if present. Detailed information on the collection and categorization of these data is presented elsewhere [21]. In addition, the Dutch comorbidity questionnaire, Statistics Netherlands, was used to assess the most common chronic diseases [23], categorized into diabetes mellitus, neurovascular disease, cardiovascular disease, and malignancies. Finally, the Short Form-Health and Labor Questionnaire (SF-HLQ) [24] was used to determine whether patients had a paid job.

For the cross-sectional cohort, data on age, sex, marital status, education level, tumor type, date of diagnosis, pituitary function, and work status were collected and categorized similarly to the longitudinal cohort [22].

#### Health-related quality of life

Patients completed the SF-36 version 1 [12], which was originally developed and validated in patients with hypertension, diabetes mellitus, congestive heart failure, myocardial infarction, and depression [25, 26]. The PCS and MCS of the SF-36 range from 0 to 100, higher scores indicating a better HRQoL. The PCS and MCS of the SF-12 were calculated using the 12 corresponding items of the SF-36 [27] and similarly range from 0 to 100, higher scores indicating a better HRQoL. The SF-12 was developed and validated in the general population of the United States and the same patient populations as the SF-36 and includes the 12 items that predicted the SF-36 subscales most accurately in these populations [27]. The Dutch versions of the SF-36 and SF-12 have been validated in the Netherlands [28, 29].

### Statistical analysis

In order to determine the correlation between SF-36 and SF-12 scores of the longitudinal cohort, intraclass correlation coefficients (ICCs) for absolute agreement were calculated between the component scores of both questionnaires at the different timepoints. Moreover, ICCs for absolute agreement were used to assess the correlation between change in SF-36 and SF-12 scores (preoperatively vs. 6 months postoperatively). An ICC value of ≥0.41 was considered fair; ICC ≥0.61 moderate; and ICC ≥0.81 substantial [30].

Bland–Altman plots [31] were created to assess agreement of the SF-12 and SF-36 scores at each timepoint. Bland–Altman plots are scatter plots, showing the differences between SF-36 and SF-12 scores for individual patients plotted against the mean of each patient’s SF-36 and SF-12 scores. In each plot, the population mean ($\overline d$) of all individual differences between the two scores is visualized, as well as the limits of agreement, which represent the 95% range of all individual measurements (calculated as $\overline d$ + 1.96 × SD_difference_ and $\overline d$ − 1.96 × SD_difference_). Similarly, Bland–Altman plots were created to assess agreement of the change in SF-12 and SF-36 scores over time (6 months vs. preoperatively).

To assess the course of HRQoL over time, proportions of patients in the following categories were calculated twice using the SF-36 items and SF-12 items: no relevant change on all timepoints, persistent improvement or deterioration (on both 6 weeks and 6 months), transient improvement or deterioration (only at 6 weeks) and late improvement or deterioration (only at 6 months). A clinically relevant change in SF-36 scores is not yet known for pituitary patients, but in chronic disease populations, 0.5 SD is typically regarded as the minimal important difference for HRQoL instruments [32]. Therefore, a clinically relevant change (improvement or deterioration) was defined as ≥0.5 SD of the change in SF-36 scores, and no relevant change as <0.5 SD.

To determine the ability of the SF-12 to replicate clinically relevant changes, the proportion of patients that had a clinically relevant change in the same direction on both the SF-36 and the SF-12 was calculated.

In order to assess whether the degree of disagreement between SF-36 and SF-12 scores was associated with specific baseline characteristics, patients were categorized into a group with large individual differences between the SF-36 and SF-12, and a group with good agreement of SF-36 and SF-12 scores (all other patients). Following the same line of reasoning as above, the cutoff for large individual differences between SF-36 and SF-12 was defined as 0.5 SD of the change in SF-36 scores. Logistic regression analysis (both crude and adjusted for age, sex, comorbidities, and education level) was used to determine the association between baseline factors and having >5 points difference between SF-36 and SF-12 scores on PCS and/or MCS.

For the cross-sectional cohort, ICCs for absolute agreement, Bland–Altman plots, and logistic regression analyses were calculated and performed similarly for the cohort’s single measurement. *P* values <0.05 were considered statistically significant. All statistical analyses were performed using IBM SPSS 25.0 software (Armonk, NY) [33].

## Results

### Patient populations and missing data

The longitudinal perioperative cohort consisted of 103 patients, with a median age of 52.9 years (interquartile range [IQR] 37.0–65.0 years), of whom 71 (62.8%) were female (Table 1). Most patients were diagnosed with a nonfunctioning adenoma (NFA) (*N* = 52, 44.8%), followed by acromegaly (*N* = 17, 14,7%), Cushing’s disease (*N* = 15, 12.9%), prolactinoma (*N* = 20, 17.2%), Rathke’s cleft cyst (RCC) (*N* = 7, 6.0%), and craniopharyngioma (*N* = 5, 4.3%). Preoperatively, SF-36 scores could be calculated for 99 patients, and SF-12 scores for 102 patients. At 6 weeks, calculation of all scores was possible for 100 patients. At 6 months, PCS36, MCS36, and MCS12 could be calculated for 96 patients, and PCS12 for 95 patients.

**Table 1:** Baseline characteristics

	Longitudinal cohort (*N* = 103)	Cross-sectional cohort (*N* = 413)
**Sociodemographic characteristics**		
Sex: female, *N* (%)	64 (62.1)	231 (55.9)
Tumor type, *N* (%)		
Nonfunctioning adenoma	47 (45.6)	167 (40.4)
Acromegaly	14 (13.6)	77 (18.6)
Cushing’s disease	15 (14.6)	45 (10.9)
Prolactinoma	16 (15.5)	116 (28.1)
Rathke’s cleft cyst	6 (5.8)	6 (1.5)
Craniopharyngioma	5 (4.9)	2 (0.5)
Age in years, median (IQR)	52.9 (37.0–65.0)	61.4 (49.8–70.1)
Marital status: relationship/married, *N* (%)	74 (71.8)	315 (76.5)
Education, *N* (%)		
Low	29 (28.2)	151 (36.7)
Intermediate	29 (28.2)	98 (23.8)
High	45 (43.7)	163 (39.6)
Comorbidities		NA
Diabetes mellitus	5 (5.0)	
Neurovascular disease	2 (2.0)	
Cardiovascular disease^a^	41 (40.6)	
Malignancies	14 (14.1)	
Paid job, *N* (%)	59 (59.0)	187 (45.3)
**Disease-specific characteristics**		
Tumor size, *N* (%)		NA
Micro	22 (21.4)	
Macro	58 (56.3)	
Giant	8 (7.8)	
Residual < 1 cm (previous surgery)	5 (4.9)	
Residual > 1 cm (previous surgery)	10 (9.7)	
Tumor invasion: Knosp grade		NA
0	30 (29.1)	
I	43 (41.7)	
II	21 (20.4)	
IIIA	3 (2.9)	
IIIB	4 (3.9)	
IV	2 (1.9)	
Time since diagnosis, in years, median (IQR)	0.8 (0.1; 4.8)	13.0 (5.7; 23.4)
Prior treatment, *N* (%)		NA
No treatment	59 (57.3)	
Medication	29 (28.2)	
Surgery	15 (14.6)	
Radiotherapy	0	
Preoperative pituitary function, *N* (%)		
No deficits	48 (46.6)	175 (42.4)
Hypopituitarism	50 (48.5)	156 (37.8)
Panhypopituitarism	5 (4.9)	82 (19.9)
Preoperative visual field status, *N* (%)		NA
No deficits	56 (54.4)	
Mild visual field deficits (quadrantanopia)	19 (18.4)	
Severe visual field deficits (hemianopia)	28 (27.2)	
Cranial nerve palsy, *N* (%)	3 (2.9)	NA

Due to rounding, not all percentages of the categorical variables add up to 100%

*N* number, *SD* standard deviation, *IQR* interquartile range, *NA* not available, because these data were not collected in the cross-sectional cohort

^a^Cardiovascular disease includes hypertension, atherosclerosis and myocardial infarction

The cross-sectional chronic care cohort consisted of 431 patients, with a median age of 61.4 years (IQR 49.8–70.1 years). Of these patients, 231 were female (55.9%). The most common tumor type was NFA (*N* = 167, 40.4%). Acromegaly was diagnosed in 77 patients (18.6%), Cushing in 45 patients (10.9%), prolactinoma in 116 patients (28.1%), RCC in six patients (1.5%), and craniopharyngioma in two patients (0.5%). SF-36 scores could be calculated for 411 patients, and SF-12 scores for 413 patients.

### Longitudinal (perioperative) SF-36 and SF-12 scores

**Fig. 1:**
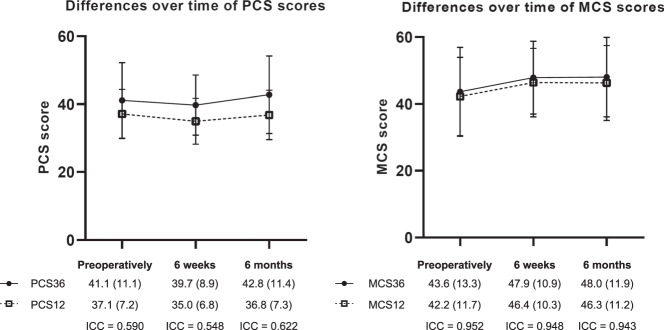
Longitudinal cohort—Mean SF-36 and SF-12 scores (SD) and intraclass correlation coefficients between SF-36 and SF-12 scores, per timepoint. SD standard deviation; ICC intraclass correlation coefficient; PCS physical component score; MCS mental component score

In the longitudinal cohort, mean PCS36 decreased from 41.4 preoperatively to 39.7 at 6 weeks and increased to 42.9 at 6 months postoperatively (Fig. 1). PCS12 scores were consistently slightly lower than PCS36 scores, with values of 37.1 preoperatively, 35.0 at 6 weeks and 36.8 at 6 months. MCS36 and MCS12 scores were more comparable, with scores of 43.5 and 42.0 preoperatively, 47.9 and 46.4 at 6 weeks, and 48.1 and 46.4 at 6 months, respectively. Scores were similar in the cross-sectional study (Supplementary 1).

### Correlation of SF-36 and SF-12

In the longitudinal cohort, the ICCs of the PCS were 0.590 preoperatively, 0.548 at 6 weeks and 0.622 at 6 months (Fig. 1), only the latter correlation being considered moderate for the majority of tumor types (Supplementary 2 and 3). On the contrary, the ICCs of the MCS were substantial at all timepoints (0.952 preoperatively, 0.948 at 6 weeks, 0.943 at 6 months) and for all tumor types (Supplementary 2 and 3). Results were similar for the cross-sectional cohort (Supplementary 4 and 5).

**Fig. 2:**
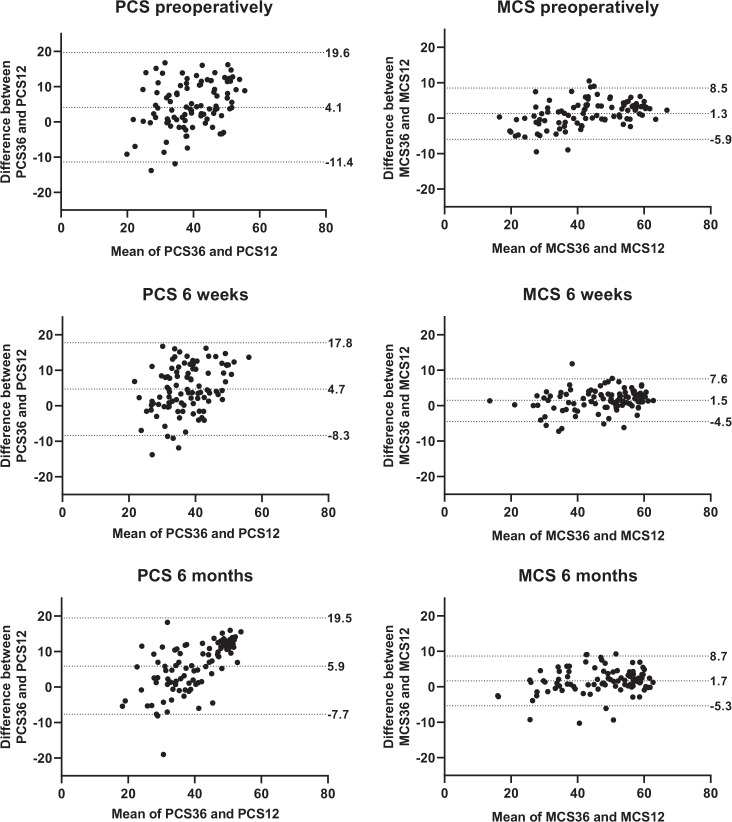
Longitudinal cohort—Mean difference and limits of agreement between SF-36 and SF-12 scores (Bland–Altman plots), per timepoint. PCS physical component score; MCS mental component score. Limits of agreement depict 95% of the individual patient differences between SF-36 and SF-12

In line with these results, the Bland–Altman plots (Fig. 2) of the PCS of the longitudinal cohort showed relatively wide limits of agreement for individual patients (−11.4 to 19.6 preoperatively; −8.3 to 17.8 at 6 weeks; −7.7 to 19.5 at 6 months), with mean differences of 4.1, 4.7, and 5.9 points respectively for the whole group, while the limits of agreement of the MCS were narrower (−5.9 to 8.5 preoperatively; −4.5 to 7.6 at 6 weeks; −5.3 to 8.7 at 6 months), with mean differences of 1.3, 1.5, and 1.7 points respectively. The Bland–Altman plots (Supplementary 6) of the cross-sectional cohort were in concordance with those of the longitudinal cohort.

### Longitudinal changes in SF-36 and SF-12

**Fig. 3:**
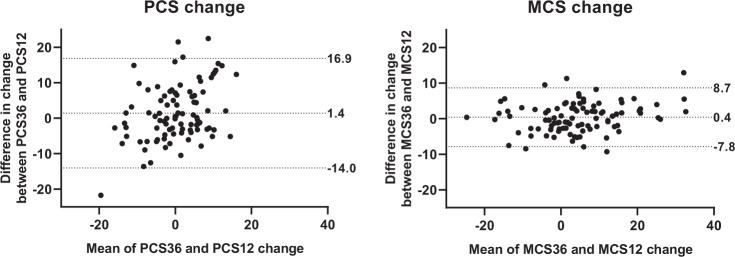
Longitudinal cohort—Mean difference and limits of agreement between SF-36 and SF-12 change in scores (Bland–Altman plots). Differences are between baseline and measurement at 6 months. PCS physical component score; MCS mental component score. Limits of agreement depict 95% of the individual patient differences between SF-36 and SF-12

In the longitudinal cohort, mean longitudinal changes (6 months vs. preoperatively) were comparable between SF-36 (PCS 1.3; MCS 4.5) and SF-12 (PCS −0.3; MCS 3.8) scores. However, the correlation for change in SF-36 and SF-12 scores was substantial only for MCS (ICC = 0.931), while the ICC for PCS was considered fair (ICC = 0.546). Limits of agreement were −14.0 to 16.9 for PCS, and −7.8 to 8.7 for MCS, with mean differences of 1.4 for PCS and 0.4 for MCS (Fig. 3). Longitudinal changes of the PCS and MCS between the preoperative measurement and 6 months postoperatively could be calculated for 94 patients for the PCS36, PCS12, and MCS36, and for 95 patients for the MCS12.

**Fig. 4:**
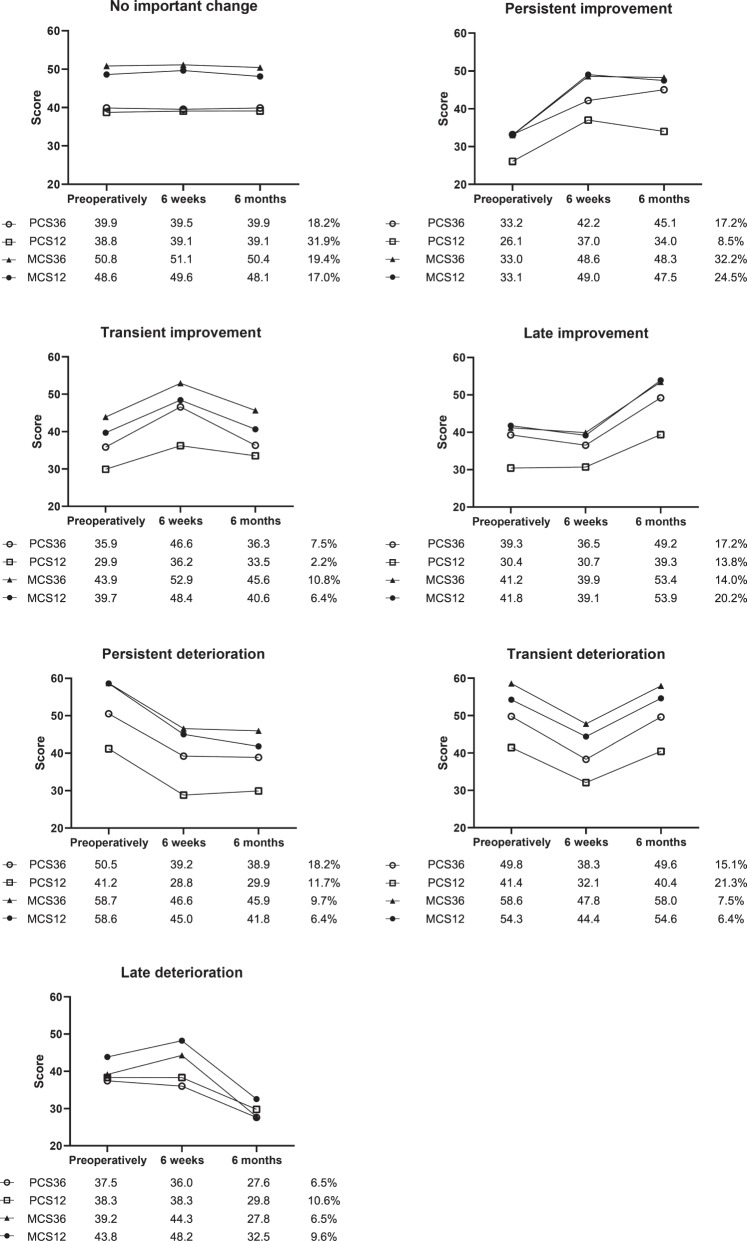
Longitudinal cohort—Course of SF-36/SF-12 scores of patient groups with no, persistent, transient, or late change on SF-36/SF-12. Percentages add up to 100% for PCS36, PCS12, MCS36, and MCS12. PCS physical component score; MCS mental component score

The SDs of the change in SF-36 scores were around 10 in this study (data not shown), and the clinically relevant change (0.5 SD) therefore approached 5. Compared with the SF-36 component scores, the PCS12 and MCS12 showed a lower proportion of patients in the clinically relevant improvement categories, and the PCS12 showed a higher proportion of patients in the deterioration categories (Fig. 4). The percentage of patients with no important change on PCS12 (31.9%) was substantially higher than the percentage with no important change on PCS36 (18.2%). Importantly, only the group without relevant change had similar SF-36 and SF-12 scores for both PCS and MCS. Moreover, the patient groups that improved over time had on average lower baseline scores than the patients that deteriorated.

Of the patients with a clinically relevant increase (>5 points) on PCS36, 37.5% also had a clinically relevant increase on PCS12 (Table 2). Of the patients with a clinically relevant decrease on PCS36, 47.8% had a clinically relevant decrease on the PCS12. The numbers for the MCS were higher, 79.1% for increase and 87.5% for decrease, respectively (Table 2).

**Table 2:** Longitudinal cohort – Proportion of patients with corresponding clinically relevant changes on SF-36 and SF-12 component scores between baseline and 6 months

Physical component score				
	PCS12			Total
PCS36	No important difference	>5 points increase	>5 points decrease	
No important difference	23 (60.5%)	8 (21.1%)	7 (18.4%)	38 (100%)
>5 points increase	17 (53.1%)	12 (37.5%)	3 (9.4%)	32 (100%)
>5 points decrease	12 (52.2%)	0	11 (47.8%)	23 (100%)

Mental component score				
	MCS12			Total
MCS36	No important difference	>5 points increase	>5 points decrease	
No important difference	26 (74.3%)	8 (22.9%)	1 (2.9%)	35 (100%)
>5 points increase	9 (20.9%)	34 (79.1%)	0	43 (100%)
>5 points decrease	2 (12.5%)	0	14 (87.5%)	16 (100%)

*PCS* physical component score, *MCS* mental component score

### Association of baseline factors with difference between SF-36 and SF-12 scores

As the minimal important difference (0.5 SD) approached 5 in this study, the cutoff for large individual differences between SF-12 and SF-36 PCS and/or MCS scores was set at 5 points.

Preoperatively, 69 patients of the longitudinal cohort (69.7%) had a large individual difference between SF-36 and SF-12. At 6 weeks, this group consisted of 59 patients (59.0%), and at 6 months of 74 patients (77.9%). In the cross-sectional cohort, 318 patients (77.4%) had a difference of >5 points between SF-36 and SF-12 scores on PCS and/or MCS. Overall, no consistent significant associations were found between baseline factors (i.e., sex, tumor type, age, education level, comorbidities, tumor size, time since diagnosis, prior treatment, preoperative pituitary function, and preoperative visual deficits) and having >5 points difference between the two questionnaires (Supplementary 7–9).

## Discussion

The present post hoc analysis of two existing cohorts of patients with a pituitary/sellar tumor demonstrates that, on a group level, the MCS derived from the SF-36 and SF-12 shows substantial agreement on all timepoints and over time. However, the agreement between the PCS of both questionnaires is less convincing, since these correlations were not more than fair in both cohorts. Moreover, due to large individual differences between SF-36 and SF-12, the SF-12 cannot reliably replace the SF-36 for individual patients.

SF-36 and SF-12 scores could be calculated for similar numbers of patients. The Bland–Altman plots demonstrated that the mean differences between the SF-36 and SF-12 scores were up to two points for the MCS, and up to six points for the PCS, indicating comparable results for the MCS between both questionnaires on a group level, when individual scores are averaged. However, the limits of agreement show that individual differences between the SF-36 of SF-12 for both the MCS and PCS are large, varying up to seven points for the MCS and up to 15 points for the PCS, which implies that the SF-12 score of an individual patient may differ up to seven (MCS) or 15 (PCS) points from their SF-36 score. Regression analysis was used to assess whether large individual differences were related to specific baseline factors, but overall, no consistently significant associations between baseline factors and a large individual disagreement between the SF-36 and SF-12 were found in both cohorts. Bland–Altman plots were also used to assess to what extent the component scores of both questionnaires showed a comparable change over time. Again, mean differences in change over time were small, but the limits of agreement were wide, varying up to 15 points (PCS), indicating that the change of the SF-12 of an individual patient may differ strongly from the change of their SF-36 scores. Importantly, the proportion of patients with a clinically relevant change in the same direction on both the SF-36 and SF-12 was as low as 37.5% for a clinically relevant increase in the PCS, while the percentages were considerably higher for the MCS.

The SF-36 and SF-12 have been compared previously in other patient groups, such as dialysis patients [14], patients undergoing knee replacement surgery [16], and patients with a history of stroke [17] (Supplementary 10). Comparable with our study (ICC range: 0.943–0.952), most other studies found good correlations between the MCS of the SF-36 and SF-12 (ICC range: 0.93–0.97). However, while we found a poor correlation for the PCS (ICC range: 0.548–0.622), most studies [14–20] also found a good correlation for this component score (ICC range: 0.92–0.97). The majority of the studies therefore concluded that the SF-12 scores reliably approach the SF-36 scores, for both the PCS and MCS [14–20]. Moreover, most longitudinal studies concluded that responsiveness to change was also comparable between the SF-36 and SF-12 [16, 18–20, 34–37], reporting correlations (*r* or ICC) for change ranging between 0.84 and 0.94 for the PCS, and between 0.90 and 0.95 for the MCS. In contrast, the present study showed that individual differences between change in SF-36 and SF-12 scores can be large, and that the ICC for change of the PCS (ICC = 0.546) was considerably lower than for the MCS (ICC = 0.931). The large discrepancy between the PCS and MCS correlations and limits of agreement found in our study is not consistent with the existing literature in other patient groups such as osteoarthritis or stroke patients [14–17, 19, 20, 34–37], and might reflect the complex multisystem morbidity of endocrinological conditions. The SF-36 and SF-12 were developed and validated in patient populations with typically less complex morbidity, such as hypertension and myocardial infarction. In pituitary patients, typically, a combination of multiple less apparent symptoms (fatigue and psychological symptoms) and symptoms that are difficult to measure may profoundly impact their HRQoL [8], requiring measurement with the more comprehensive SF-36 instead of the SF-12. For instance, as pituitary patients experience limitations in energy rather than function, it can be expected that physical HRQoL impairment will be reflected by limitations in moderate activities (included in the SF-12), rather than by limitations in light activities such as walking one block or dressing oneself (not included in the SF-12). Indeed, as outlined in Supplementary 11, the SF-12 includes the physical SF-36 items that in general score relatively low in this cohort, while the items not included in the SF-12 score higher. This may in part explain the marked discrepancy between PCS scores of the two questionnaires. Notably, disease-specific characteristics influence the comparability of the SF-36 and SF-12, and therefore, it is important to evaluate per condition whether this shortened version is representative.

Besides the SF-36, other brief generic questionnaires such as the EuroQoL-5D [38] have been used in pituitary patients [39–41]. However, this widely used questionnaire only consists of five items, limiting its ability to provide a comprehensive view on the self-perceived health of patients with complex conditions such as pituitary diseases. This is partially depicted by a strong ceiling effect, as most patients report (very) high scores and therefore, most patients only have room for deterioration [21]. Moreover, the EuroQoL-5D is primarily a questionnaire assessing utility, which is used for economic evaluations and should be distinguished from HRQoL. The SF-36 is therefore more suitable, as a generic HRQoL instrument, for individual patient care than the EuroQoL-5D.

### Strengths and limitations

Strengths of this study include the use of two cohorts, thereby increasing patient numbers and allowing for not only cross-sectional analysis in a chronic care setting, but also longitudinal analysis in a perioperative setting. Furthermore, the patient population included in the study is heterogeneous and conclusions can therefore be generalized to the total pituitary patient population. Regression analysis showed that this heterogeneity has not influenced the study’s outcomes.

A few limitations of this study must be noted. First of all, in the cohorts used in this analysis, the SF-12 was not assessed separately, but was calculated from the SF-36. This may have resulted in slightly different SF-12 scores than would have been obtained using the SF-12 questionnaire. However, in previous research SF-12 scores based on the items embedded in the SF-36 were found to be equivalent to the scores obtained when the SF-12 was administered separately [42]. Furthermore, although the SF-12 and SF-36 have been validated in several countries, differences between and within both questionnaires scores may exist between countries [28, 43], possibly resulting in a limited generalizability of the results of this study.

## Conclusions

PROMs are increasingly used in both clinical trials and clinical practice. In clinical trials, PROMs serve as HRQoL outcome measures [44–46], that consequently influence clinical decision making, health care policy [47], and guideline development [48, 49]. In clinical practice, PROMs enable patient monitoring and facilitate patient–doctor communication [50], resulting in the identification of previously unrecognized symptoms, and improvement of patient satisfaction and outcomes [51–54]. Our research team has obtained experience with a combination of several PROMs in a comprehensive outcome set for pituitary care [21], which harmonizes outcomes, and enables systematic assessment of HRQoL of all patients. However, this comprehensive outcome set can be time-consuming and therefore burdensome for patients, due to the relatively large number of questions [21]. The present study therefore investigated whether the shorter SF-12 can be used instead of the SF-36 in patients with pituitary/sellar disease and showed that on a group level, the SF-12 can indeed reliably replicate the MCS, whereas evidence for the PCS is less convincing. However, due to large individual differences between SF-36 and SF-12 scores, the SF-12 is not suitable to replicate SF-36 scores for individuals in this population. Given the additional advantage of the SF-36 of generating domain scores, which provide clinicians and nurses with quick insight into the different aspects of patients’ HRQoL, we recommend the SF-36 for clinical use in individual pituitary patients. Whether the SF-12 may fulfill the requirements of a generic PROM in the comprehensive set of generic, disease-specific, and symptom-specific PROMs for pituitary patients needs to be evaluated. In the meantime, alternative approaches to decrease the number of questions in this comprehensive outcome set, such as computer adaptive testing [55–57], should be explored as well.

## Supplementary information

Supplementary Information

## Data Availability

Data requests can be directed to D.J.L.
