# Supplementary material for: SF-12 or SF-36 in pituitary disease? Toward concise and comprehensive patient-reported outcomes measurements
Source: Endocrine. 2020 Jun 19;70(1):123–33. doi: 10.1007/s12020-020-02384-4 (PMC7525280; doi:10.1007/s12020-020-02384-4)
Supplement: Supplementary file 1 — Supplementary Information [file 12020_2020_2384_MOESM1_ESM.pdf]

Supplements

Supplement 1. ***Cross-sectional cohort*** *– Mean SF-36 and SF-12 scores*

	Mean (SD)	N
**PCS36**	45.0 (10.7)	411
**PCS12**	38.8 (6.9)	413
**MCS36**	49.7 (11.4)	411
**MCS12**	47.8 (10.2)	413

SD, standard deviation; N, number; PCS, physical component score; MCS, mental component score.

Supplement 2. ***Longitudinal cohort*** *– Intraclass correlation coefficients between SF-36 and SF-12 scores, per timepoint and per tumor type*

	Preop	P-value	N	6 weeks	P-value	N	6 months	P-value	N
**PCS**									
Total	0.590	0.000	99	0.548	0.000	100	0.622	0.000	95
NFA	0.473	0.000	46	0.512	0.000	46	0.621	0.000	43
ACRO	0.613	0.031	13	0.685	0.000	14	0.617	0.000	13
CD	0.585	0.008	14	0.416	0.156	14	0.714	0.000	14
PRL	0.684	0.000	15	0.609	0.005	16	0.382	0.052	15
RCC	0.541	0.224	6	0.199	0.652	6	0.843	0.001	6
Cranio	0.363	0.227	5	0.720	0.017	4	-0.107	0.754	4
**MCS**									
Total	0.952	0.000	99	0.948	0.000	100	0.943	0.000	96
NFA	0.955	0.000	46	0.949	0.000	46	0.911	0.000	43
ACRO	0.849	0.000	13	0.948	0.000	14	0.895	0.000	13
CD	0.944	0.000	14	0.939	0.000	14	0.969	0.000	14
PRL	0.935	0.000	15	0.959	0.000	16	0.944	0.000	15
RCC	0.946	0.000	6	0.853	0.014	6	0.917	0.001	6
Cranio	0.973	0.007	5	0.943	0.038	4	0.982	0.002	5

Preop, preoperatively; N, number; PCS, physical component score; MCS, mental component score; NFA, non-functioning adenoma; ACRO, acromegaly; CD, Cushing's disease; PRL, prolactinoma; RCC, Rathke's cleft cyst; Cranio, craniopharyngioma.

Supplement 3. ***Longitudinal cohort*** *– Intraclass correlation plots of SF-36 and SF-12 scores, per timepoint*

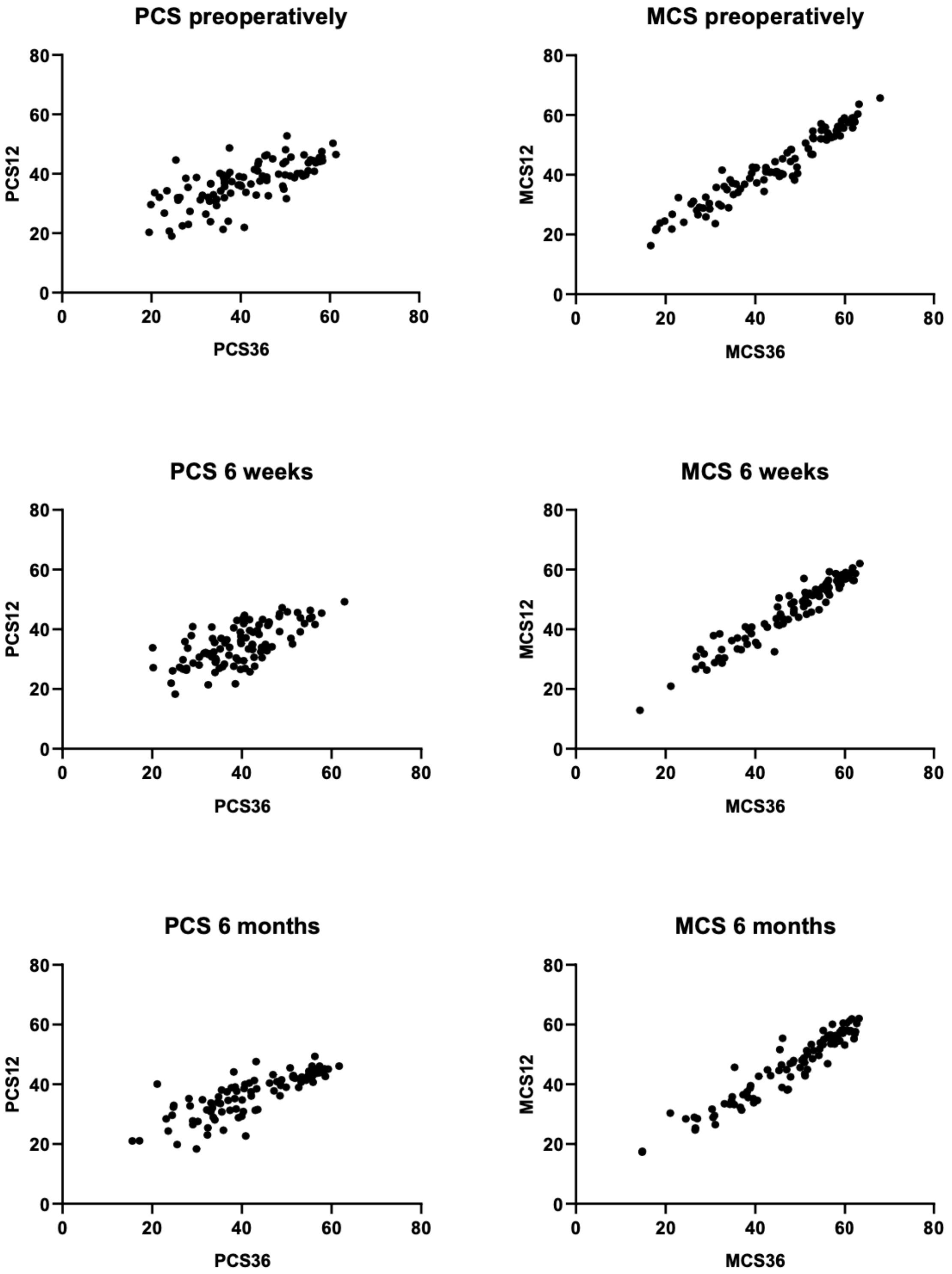

PCS, physical component score; MCS, mental component score.

Supplement 4. ***Cross-sectional cohort*** *– Intraclass correlation coefficients (Pearson) between SF-36 and SF-12 scores, per tumor type*

	**ICC**	**N**	**P-value**
**PCS**			
**Total**	0.597	411	0.000
**Non-functioning adenoma**	0.625	165	0.000
**Acromegaly**	0.656	77	0.000
**Cushing's disease**	0.521	45	0.000
**Prolactinoma**	0.483	116	0.000
**RCC/Craniopharyngioma**	0.718	8	0.011
**MCS**			
**Total**	0.943	411	0.000
**Non-functioning adenoma**	0.940	165	0.000
**Acromegaly**	0.924	77	0.000
**Cushing's disease**	0.955	45	0.000
**Prolactinoma**	0.954	116	0.000
**RCC/Craniopharyngioma**	0.945	8	0.000

ICC, intraclass correlation coefficient; N, number; PCS, physical component score; MCS, mental component score; RCC, Rathke's cleft cyst.

Supplement 5. ***Cross-sectional cohort*** *– Intraclass correlation plots of SF-36 and SF-12 scores*

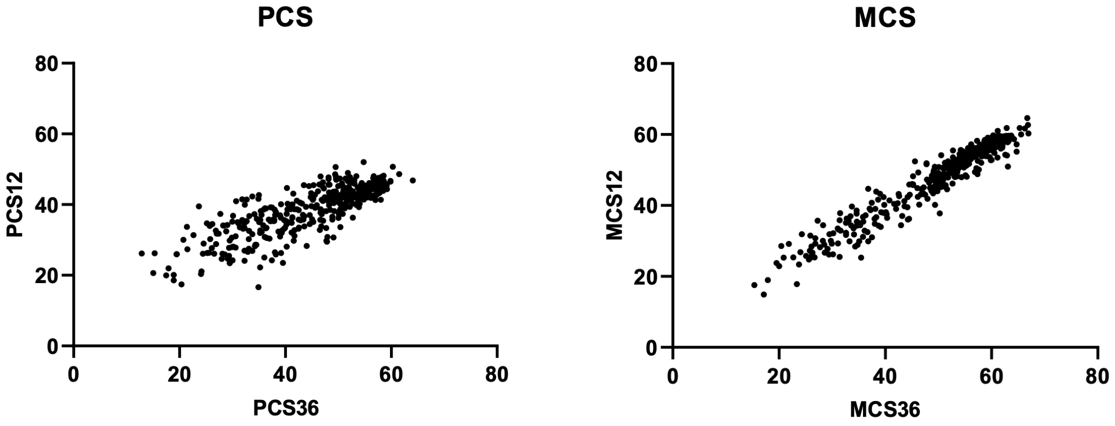

PCS, physical component score; MCS, mental component score.

Supplement 6. ***Cross-sectional cohort*** *– Mean difference and limits of agreement between SF-36 and SF-12 scores (Bland-Altman plots)*

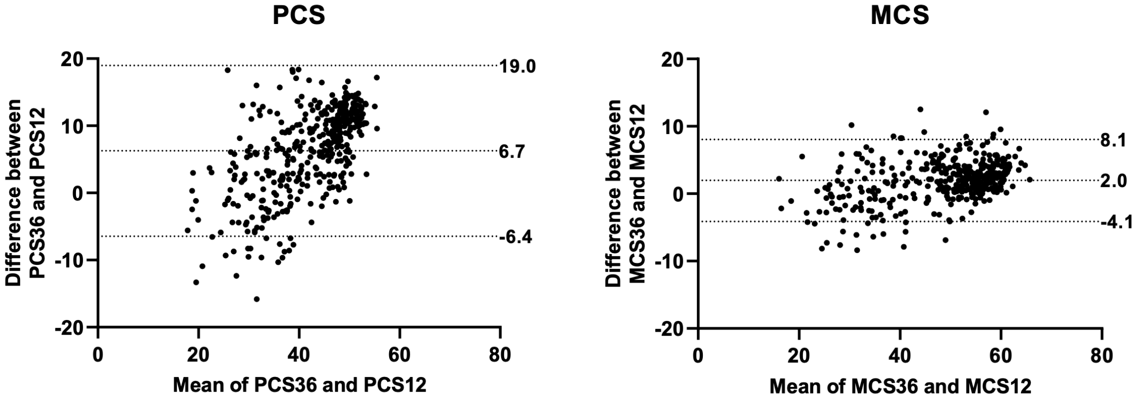

PCS, physical component score; MCS, mental component score.

Supplement 7. ***Longitudinal cohort*** *– Association of baseline factors with a minimum of 5 points difference between SF-36 and SF-12 scores: univariable logistic regression analyses at all three timepoints*

	**Preoperatively** (N = 99, missing = 4)		**6 weeks** (N = 100, missing = 3)		**6 months** (N = 95, missing = 8)	
	OR	95% CI	OR	95% CI	OR	95% CI
**Sex**: female	0.591	0.237; 1.477	0.903	0.396; 2.057	0.345	0.106; 1.127
**Tumor type** (reference: NFA)						
Acromegaly	0.412	0.115; 1.472	0.704	0.212; 2.338	0.211	0.049; 0.902
Cushing's disease	2.118	0.413; 10.865	0.938	0.280; 3.147	0.237	0.056; 0.996
Prolactinoma	0.403	0.120; 1.352	1.173	0.364; 3.779	0.526	0.109; 2.534
Rathke's cleft cyst	0.706	0.114; 4.359	1.407	0.234; 8.480	0.263	0.038; 1.825
Craniopharyngioma	1.412	0.143; 13.913	2.111	0.204; 21.873	0.395	0.034; 4.564
**Age** in years	0.992	0.967; 1.018	1.025	**1.001; 1.050**	1.023	0.993; 1.054
**Education level** (reference: Low)						
Intermediate	1.765	0.554; 5.620	1.133	0.393; 3.269	1.111	0.345; 3.575
High	1.403	0.509; 3.869	1.271	0.481; 3.358	3.200	0.915; 11.196
**Comorbidities**: yes						
Diabetes mellitus	0.249	0.039; 1.578	0.421	0.067; 2.644	0.386	0.060; 2.484
Neurovascular disease	0.397	0.024; 6.578	0.655	0.040; 10.794	0.000	0.000; -
Cardiovascular disease	1.118	0.459; 2.723	1.225	0.538; 2.789	1.081	0.395; 2.959
Malignancies	0.290	**0.088; 0.961**	1.249	0.385; 4.054	1.022	0.256; 4.083
**Tumor size** (reference: Micro)						
Macro	0.735	0.245; 2.201	1.067	0.397; 2.868	2.611	0.823; 8.280
Giant	$6.9*10^8$	0.000; -	5.000	0.513; 48.750	$8.1*10^8$	0.000; -
Residual <1 cm	1.714	0.157; 18.726	1.250	0.173; 9.019	0.500	0.058; 4.335
Residual >1 cm	1.714	0.278; 10.589	1.667	0.330; 8.423	0.833	0.153; 4.537
**Time since diagnosis** in years	0.969	0.876; 1.071	1.042	0.941; 1.154	0.969	0.853; 1.101
**Prior treatment** (reference: None)						
Medication	0.722	0.275; 1.897	0.833	0.337; 2.054	0.359	0.121; 1.070
Surgery	1.700	0.425; 6.802	1.218	0.361; 4.102	0.340	0.083; 1.401
**Preoperative pituitary function** (reference: No deficits)						
Hypopituitarism	1.600	0.656; 3.902	1.363	0.602; 3.087	1.588	0.578; 4.360
Panhypopituitarism	0.800	0.121; 5.292	3.385	0.352; 32.554	1.412	0.143; 13.913
**Preoperative visual deficits** (reference: No deficits)						
Mild deficits	0.317	**0.104; 0.967**	1.869	0.585; 5.975	$7.8*10^8$	0.000; -
Severe deficits	0.753	0.267; 2.120	0.774	0.307; 1.953	2.671	0.794; 8.983

OR, odds ratio; 95% CI, 95% confidence interval; NFA, non-functioning adenoma.

Supplement 8. ***Longitudinal cohort*** *– Association of baseline factors with difference between SF-36 and SF-12 scores: univariable logistic regression analysis, adjusted for age, sex, education, and comorbidities*

	**Preoperatively** (N = 99, missing = 4)		**6 weeks** (N = 100, missing = 3)		**6 months** (N = 95, missing = 8)	
	OR	95% CI	OR	95% CI	OR	95% CI
**Sex**: female	0.680	0.243; 1.907	1.130	0.455; 2.807	0.332	0.089; 1.235
**Tumor type** (reference: NFA)						
Acromegaly	0.150	**0.030; 0.756**	0.673	0.175; 2.587	0.036	**0.004; 0.322**
Cushing's disease	1.155	0.175; 7.621	1.226	0.293; 5.125	0.085	**0.009; 0.795**
Prolactinoma	0.094	**0.015; 0.592**	2.711	0.605; 12.155	0.612	0.066; 5.653
Rathke's cleft cyst	1.018	0.112; 9.263	1.365	0.195; 9.574	0.091	0.007; 1.128
Craniopharyngioma	0.499	0.038; 6.629	2.867	0.233; 35.314	0.085	0.004; 1.662
**Age** in years	0.998	0.966; 1.030	1.031	**1.000; 1.062**	1.024	0.986; 1.063
**Education level** (reference: Low)						
Intermediate	1.241	0.327; 4.709	1.552	0.474; 5.077	1.323	0.343; 5.106
High	0.841	0.353; 3.593	1.404	0.477; 4.133	3.896	0.893; 16.996
**Comorbidities**: yes						
Diabetes mellitus	0.123	**0.015; 0.981**	0.349	0.048; 2.563	0.224	0.023; 2.167
Neurovascular disease	0.296	0.014; 6.097	0.405	0.020; 8.171	0.000	0.000; -
Cardiovascular disease	1.739	0.590; 5.121	1.105	0.438; 2.785	1.467	0.421; 5.113
Malignancies	0.262	0.063; 1.094	0.700	0.169; 2.899	0.812	0.146; 4.502
**Tumor size** (reference: Micro)						
Macro	1.078	0.290; 4.011	1.026	0.327; 3.215	6.940	**1.210; 39.804**
Giant	$1.7*10^9$	0.000; -	5.911	0.426; 82.068	$2.0*10^9$	0.000; -
Residual <1 cm	2.366	0.162; 34.632	1.135	0.139; 9.263	0.184	0.010; 3.513
Residual >1 cm	3.593	0.309; 41.719	1.967	0.285; 13.594	0.209	0.018; 2.485
**Time since diagnosis** in years	0.929	0.830; 1.040	1.040	0.931; 1.162	0.916	0.785; 1.069
**Prior treatment** (reference: None)						
Medication	0.502	0.146; 1.732	1.154	0.385; 3.459	0.240	0.054; 1.064
Surgery	1.907	0.342; 10.644	1.289	0.316; 5.257	0.036	**0.004; 0.321**
**Preoperative pituitary function** (reference: No deficits)						
Hypopituitarism	2.915	0.899; 9.447	1.090	0.396; 3.001	1.336	0.373; 4.785
Panhypopituitarism	1.088	0.130; 9.082	2.429	0.223; 26.480	0.806	0.061; 10.635
**Preoperative visual deficits** (reference: No deficits)						
Mild deficits	0.303	0.088; 1.043	1.399	0.401; 4.884	$8.9*10^8$	0.000; -
Severe deficits	1.126	0.305; 4.154	0.657	0.215; 2.002	4.662	0.889; 24.447

N, number; OR, odds ratio; 95% CI, 95% confidence interval; NFA, non-functioning adenoma.

Supplement 9. ***Cross-sectional cohort*** *– Association of baseline factors with difference between SF-36 and SF-12 scores: univariable logistic regression analysis, crude and adjusted for age, sex, and education level (N = 411, missing = 2)*

	**Crude**		**Adjusted**	
	OR	95% CI	OR	95% CI
**Age** in years	0.988	0.972; 1.005	0.988	0.971; 1.005
**Sex**: female	0.846	0.529; 1.351	0.781	0.480; 1.268
**Tumor type** (reference: NFA)				
Acromegaly	0.909	0.497; 1.661	0.858	0.462; 1.591
Cushing's disease	1.455	0.648; 3.263	1.619	0.695; 3.769
Prolactinoma	**2.273**	**1.210; 4.270**	**2.270**	**1.130; 4.559**
Rathke's cleft cyst/Craniopharyngioma	1.091	0.212; 5.607	1.246	0.234; 6.626
**Pituitary function** (ref: No deficits)				
Hypopituitarism	0.649	0.387; 1.086	0.653	0.387; 1.100
Panhypopituitarism	0.908	0.473; 1.744	0.880	0.448; 1.730
**Education level** (ref: Low)				
Intermediate	1.056	0.585; 1.904	0.976	0.536; 1.780
High	1.458	0.852; 2.495	1.319	0.757; 2.299
**Time since diagnosis** in years	1.001	0.982; 1.021	1.008	0.987; 1.029

N, number; OR, odds ratio; 95% CI, 95% confidence interval; NFA, non-functioning adenoma.

Supplement 10. Literature comparing SF-36 and SF-12 in other patient populations

Authors	Population	Cross-sectional outcomes	Longitudinal outcomes
Loosman et al. (2015) [14]	Dialysis patients (N=1379)	ICC baseline: PCS 0.92; MCS 0.94.<br>Mean difference between SF-36/SF-12: PCS 0.6; MCS -1.6.<br>Limits of agreement: PCS −6.8 to 8.0; MCS −8.7 to 5.5.	ICC for change in scores: PCS 0.84; MCS 0.90.<br>Mean difference of change between SF-36/SF-12: PCS 0.2; MCS −0.1.<br>Limits of agreement for change: PCS −8.8 to 9.2; MCS −8.3 to 8.4.
Wukich et al. (2016) [15]	Diabetic foot disease (N=300)	ICC: PCS 0.937; MCS 0.955.	
Webster et al. (2016) [16]	Patients with knee osteoarthritis and replacement surgery (N=407)	*r* preoperative: PCS 0.90; MCS 0.96.<br>*r* postoperative: PCS 0.92; MCS 0.93.	Mean change over 12 months (SD): PCS36 12.0 (8.7), PCS12 12.0 (9.3), MCS36 2.9 (10.7), MCS12 2.2 (10.9).<br>*r* of change: PCS 0.88; MCS 0.93.<br>SRMs total patient group: PCS36 1.4, PCS12 1.3; MCS36 0.3; MCS12 0.
Pickard et al. (1999) [17]	Stroke patients (N=161)	ICC self-assessment: PCS 0.959; MCS 0.954.<br>ICC proxy-assessment: PCS 0.973; MCS 0.973.	
Riddle et al. (2001) [18]	Low back pain (N=101)	*r*: PCS 0.92.<br>MCS not measured.	Mean change (SD): PCS36 11.4 (10.1), PCS12 9.9 (10.2).<br>*r* of change: PCS 0.91.<br>Area under the curve (AUC) of ROC curve: PCS36 0.78; PCS12 0.75; no significant difference ($z = 0.52$, $p_2 = 0.388$).<br>MANCOVA comparing sensitivity of change of PCS36 and PCS12: not significant ($F_{1,97} = 1.01$; $P = 0.318$).<br>MCS not measured.
Kiely et al. (2006) [19]	Non-neurological blunt trauma (N=196)	Pearson's r: PCS 0.924; MCS 0.925.	SRMs: PCS36 0.67; PCS12 0.52; MCS36 0.05; MCS12 0.02.
Müller-Nordhorn et al. (2004) [20]	Coronary heart disease (N=2441)	*r*: PCS 0.96; MCS 0.96	*r* of change: PCS 0.94; MCS 0.95.<br>SRMs:<br>- Myocardial infarction: PCS36 -0.15; PCS12 -0.18; MCS36 -0.04; MCS12 -0.05.<br>- CABG: PCS36 0.60; PCS12 0.63; MCS36 0.33; MCS12 0.37.<br>- PTCA: PCS36 0.47; PCS12 0.48; MCS36 0.25; MCS12 0.29.

Jenkinson et al. (1997) [34]	CHF (N=61); sleep apnea (N=63); inguinal hernia (N=135)		ES:<br>- CHF: PCS36 0.04; PCS12 0.01; MCS36 0.10; MCS12 0.11<br>- Sleep apnea: PCS36 0.58; PCS12 0.61; MCS36 0.81; MCS12 0.77<br>- Hernia, open surgery: PCS36 -2.00; PCS12 -2.14; MCS36 -1.13; MCS12 -1.17<br>- Hernia, laparoscopic surgery: PCS36 -0.77; PCS12 -0.83; MCS36 -0.67; MCS12 -0.66
Rubenach et al. (2002) [35]	Myocardial infarction (N=65)		SRMs at 1, 6, 12, and 24 weeks, respectively:<br>- PCS12: -0.88, -0.58, -0.42, -0.39<br>- MCS12: -0.32, -0.44, -0.33, -0.42<br>SF-36 not measured.
Bessette et al. (1998) [36]	Carpal tunnel syndrome (N=196)		Mean change over 6 months (SD): PCS36 5.33 (10.11), PCS12 6.86 (11.92), MCS36 0,19 (12.32), MCS12 0.92 (11.83).<br>SRMs: PCS36 0.53, PCS12 0.58; MCS36 0.02; MCS12 0.08.<br>ES: PCS36 0.64, PCS12 0.81; MCS36 0.02; MCS12 0.08.
Singh et al. (2006) [37]	Cervical spondylotic myelopathy (N=105)	*r* = PCS 0.94 (baseline), 0.97 (6 months); MCS 0.93 (baseline), 0.96 (6 months)	*r* of change: PCS 0.92, MCS 0.93.<br>Mean change over 6 months (SD): PCS36 14.0 (2); PCS12 14.5 (2); MCS36 14.9 (2); MCS12 16.6 (2).<br>SRM: PCS36 0.73; PCS12 0.64; MCS36 0.80; MCS12 0.75.

N, number; PCS, physical component score; MCS, mental component score; ICC, intraclass correlation coefficient; SD, standard deviation; SRM, standardized response mean (mean change/SD of change); ES, effect size (mean change/SD of baseline score); CHF, congestive heart failure; CABG, coronary artery bypass grafting; PTCA, percutaneous transluminal coronary angioplasty.

Supplement 11. Individual items of the SF-36 and SF-12, with scores at baseline in the longitudinal cohort

SF-12	SF-36	Domain	Question	Score, mean	Range of possible scores	Rescaled score (range 0-100), mean
**1**	**1**	**GH1**	**In general, would you say your health is…**	**2.88**	**1-5**	**47**
	2	HT	Compared to one year ago, how would you rate your health in general now?	3.62	1-5	66
	3	PF1	Does your health now limit you in vigorous activities, such as running, lifting heavy objects, participating in strenuous sports	1.71	1-3	36
**2**	**4**	**PF2**	**Does your health now limit you in moderate activities, such as moving a table, pushing a vacuum cleaner, bowling, or playing golf?**	**2.23**	**1-3**	**62**
	5	PF3	Does your health now limit you in lifting or carrying groceries?	2.43	1-3	72
**3**	**6**	**PF4**	**Does your health now limit you in climbing several flights of stairs**	**2.35**	**1-3**	**68**
	7	PF5	Does your health now limit you in climbing one flight of stairs	2.61	1-3	81
	8	PF6	Does your health now limit you in bending, kneeling, or stooping	2.36	1-3	68
	9	PF7	Does your health now limit you in walking more than a mile	2.31	1-3	66
	10	PF8	Does your health now limit you in walking several blocks	2.63	1-3	82
	11	PF9	Does your health now limit you in walking one block	2.78	1-3	89
	12	PF10	Does your health now limit you in bathing or dressing yourself	2.84	1-3	92
	13	RP1	During the past 4 weeks, have you had any of the following problems with your work or other regular daily activities as a result of your physical health? Cut down the amount of time you spent on work or other activities	1.42	1-2	42
**4**	**14**	**RP2**	**During the past 4 weeks, have you had any of the following problems with your work or other regular daily activities as a result of your physical health? Accomplished less than you would like**	**1.34**	**1-2**	**34**
**5**	**15**	**RP3**	**During the past 4 weeks, have you had any of the following problems with your work or other regular daily activities as a result of your physical health? Were limited in the kind of work or other activities**	**1.38**	**1-2**	**38**
	16	RP4	During the past 4 weeks, have you had any of the following problems with your work or other regular daily activities as a result of your physical health? Had difficulty performing the work or other activities (for example, it took extra effort)	1.32	1-2	32
	17	RE1	During the past 4 weeks, have you had any of the following problems with your work or other regular daily activities as a result of any emotional problems (such as feeling depressed or anxious)? Cut down the amount of time you spent on work or other activities	1.54	1-2	54

**6**	**18**	**RE2**	**During the past 4 weeks, have you had any of the following problems with your work or other regular daily activities as a result of any emotional problems (such as feeling depressed or anxious)? Accomplished less than you would like**	**1.49**	**1-2**	**49**
**7**	**19**	**RE3**	**During the past 4 weeks, have you had any of the following problems with your work or other regular daily activities as a result of any emotional problems (such as feeling depressed or anxious)? Didn't do work or other activities as carefully as usual**	**1.51**	**1-2**	**51**
	20	RE4/ SF1	During the past 4 weeks, to what extent has your physical health or emotional problems interfered with your normal social activities with family, friends, neighbors, or groups?	3.69	1-5	67
	21	BP1	How much bodily pain have you had during the past 4 weeks?	4.28	1-6	66
**8**	**22**	**BP2**	**During the past 4 weeks, how much did pain interfere with your normal work (including both work outside the home and housework)?**	**3.92**	**1-5**	**73**
	23	VT1	Did you feel full of pep?	3.93	1-6	59
	24	MH1	Have you been a very nervous person?	4.23	1-6	65
	25	MH2	Have you felt so down in the dumps that nothing could cheer you up?	5.01	1-6	80
**9**	**26**	**MH3**	**How much of the time during the past 4 weeks have you felt calm and peaceful?**	**3.77**	**1-6**	**55**
**10**	**27**	**VT2**	**How much of the time during the past 4 weeks did you have a lot of energy?**	**3.04**	**1-6**	**41**
**11**	**28**	**MH4**	**How much of the time during the past 4 weeks have you felt downhearted and blue?**	**4.50**	**1-6**	**70**
	29	VT3	Did you feel worn out?	3.76	1-6	55
	30	MH5	Have you been a happy person?	4.25	1-6	65
	31	VT4	Did you feel tired?	3.00	1-6	40
**12**	**32**	**SF2**	**During the past 4 weeks, how much of the time has your physical health or emotional problems interfered with your social activities (like visiting with friends, relatives, etc.)?**	**3.24**	**1-5**	**56**
	33	GH2	I seem to get sick a little easier than other people	3.58	1-5	65
	34	GH3	I am as healthy as anybody I know	2.90	1-5	48
	35	GH4	I expect my health to get worse	3.45	1-5	61
	36	GH5	My health is excellent	2.66	1-5	42

SF-12 (Short Form-12), SF-36 (Short Form-36), GH (general health perceptions), HT (health change), PF (physical functioning), RP (physical role functioning), RE (emotional role functioning), SF (social role functioning), BP (bodily pain), VT (vitality), MH (mental health).

Scores were calculated in such a way that a higher score means better perceived health.
In order to facilitate comparability of the scores of individual items, a rescaled score was calculated as follows: (actual score – 1) / (maximum of score range – 1) * 100.
Items in bold are included in the SF-12.

Supplement 12. STROBE Statement – Checklist of items that should be included in reports of cohort studies

	Item No	Recommendation	Where to be found
**Title and abstract**	1	(*a*) Indicate the study's design with a commonly used term in the title or the abstract	Abstract: Methods
		(*b*) Provide in the abstract an informative and balanced summary of what was done and what was found	Abstract: Results
**Introduction**			
Background/ rationale	2	Explain the scientific background and rationale for the investigation being reported	Introduction
Objectives	3	State specific objectives, including any prespecified hypotheses	Introduction
**Methods**			
Study design	4	Present key elements of study design early in the paper	Study design
Setting	5	Describe the setting, locations, and relevant dates, including periods of recruitment, exposure, follow-up, and data collection	Study design
Participants	6	(*a*) Give the eligibility criteria, and the sources and methods of selection of participants. Describe methods of follow-up	Patient population
		(*b*) For matched studies, give matching criteria and number of exposed and unexposed	*Not applicable*
Variables	7	Clearly define all outcomes, exposures, predictors, potential confounders, and effect modifiers. Give diagnostic criteria, if applicable	Data collection
Data sources/ measurement	8	For each variable of interest, give sources of data and details of methods of assessment (measurement). Describe comparability of assessment methods if there is more than one group	Data collection
Bias	9	Describe any efforts to address potential sources of bias	Reference 21 and 22
Study size	10	Explain how the study size was arrived at	Reference 21 and 22
Quantitative variables	11	Explain how quantitative variables were handled in the analyses. If applicable, describe which groupings were chosen and why	Statistical analysis
Statistical methods	12	(*a*) Describe all statistical methods, including those used to control for confounding	Statistical analysis
		(*b*) Describe any methods used to examine subgroups and interactions	
		(*c*) Explain how missing data were addressed	
		(*d*) If applicable, explain how loss to follow-up was addressed	
		(*e*) Describe any sensitivity analyses	
**Results**			
Participants	13	(a) Report numbers of individuals at each stage of study – e.g. numbers potentially eligible, examined for eligibility, confirmed eligible, included in the study, completing follow-up, and analyzed	Reference 21 and 22
		(b) Give reasons for non-participation at each stage	
		(c) Consider use of a flow diagram	

Descriptive data	14	(a) Give characteristics of study participants (e.g. demographic, clinical, social) and information on exposures and potential confounders	Patient population and missing data
		(b) Indicate number of participants with missing data for each variable of interest	
		(c) Summarize follow-up time (e.g., average and total amount)	Methods: Study design
Outcome data	15*	Report numbers of outcome events or summary measures over time	Results
Main results	16	(*a*) Give unadjusted estimates and, if applicable, confounder-adjusted estimates and their precision (e.g., 95% confidence interval). Make clear which confounders were adjusted for and why they were included	Results
		(*b*) Report category boundaries when continuous variables were categorized	Results: Longitudinal changes in SF-36 and SF-12
		(*c*) If relevant, consider translating estimates of relative risk into absolute risk for a meaningful time period	*Not applicable*
Other analyses	17	Report other analyses done – e.g. analyses of subgroups and interactions, and sensitivity analyses	Results
**Discussion**			
Key results	18	Summarize key results with reference to study objectives	Discussion (main part)
Limitations	19	Discuss limitations of the study, taking into account sources of potential bias or imprecision. Discuss both direction and magnitude of any potential bias	Discussion: Strengths and limitations
Interpretation	20	Give a cautious overall interpretation of results considering objectives, limitations, multiplicity of analyses, results from similar studies, and other relevant evidence	Discussion (main part)
Generalizability	21	Discuss the generalizability (external validity) of the study results	Discussion: Strengths and limitations
**Other information**			
Funding	22	Give the source of funding and the role of the funders for the present study and, if applicable, for the original study on which the present article is based	Funding
